# Hexokinase 2 Depletion Confers Sensitization to Metformin and Inhibits Glycolysis in Lung Squamous Cell Carcinoma

**DOI:** 10.3389/fonc.2020.00052

**Published:** 2020-01-31

**Authors:** Wenzheng Guo, Yanbin Kuang, Jingjing Wu, Donghua Wen, Aiping Zhou, Yueling Liao, Hongyong Song, Dongliang Xu, Tong Wang, Bo Jing, Kaimi Li, Min Hu, Jing Ling, Qi Wang, Wenjuan Wu

**Affiliations:** ^1^Department of Laboratory Medicine, Shanghai East Hospital, Tongji University School of Medicine, Shanghai, China; ^2^Department of Respiratory Medicine, The Second Affiliated Hospital, Dalian Medical University, Dalian, China; ^3^Department of Respiratory Medicine, School of Medicine, Ren Ji Hospital, Shanghai Jiao Tong University, Shanghai, China; ^4^Key Laboratory of Cell Differentiation and Apoptosis of Chinese Minister of Education, Shanghai General Hospital, Shanghai Jiao Tong University School of Medicine, Shanghai, China

**Keywords:** hexokinase 2, glycolysis, metformin, lung squamous cell carcinoma, apoptosis

## Abstract

Lung squamous cell carcinomas (SCCs) are highly aggressive tumors, and there is currently no effective targeted therapy owing to the lack of specific mutation targets. Compared with lung adenocarcinoma (ADCs), lung SCCs reportedly utilized higher levels of glucose metabolism to meet the anabolic and catabolic needs required to sustain rapid tumor growth. Hexokinase 2 (HK2) is an enzyme that catalyzes the rate-limit and first committed step in glucose metabolism. Here, we investigated the expression and effect of HK2 in lung SCCs. We found a significantly higher HK2 expression in lung SCCs, but not lung ADC or normal tissues. HK2 depletion or inhibition decreased the glycolysis and tumor growth via activating AMPK signaling pathway, which downregulated mTORC1 activity. Furthermore, we found an increased oxygen respiration rate compensating for HK2 depletion. Thus, metformin treatment showed combinatorial therapeutic value, which resulted in greater induction of lung SCC apoptosis *in vitro* and *in vivo*. Our study suggests that HK2 depletion in combination with metformin might be a novel effective strategy for lung SCCs therapy.

## Introduction

Lung cancer is a leading cause of cancer-related deaths worldwide, and most patients with lung cancer are diagnosed at advanced stages (1, 2). A significant increase in the morbidity and mortality of lung cancer has been reported in the last 50 years. Approximately 80% of all lung cancers are non-small-cell lung cancer (NSCLC), and squamous cell carcinoma (SCC) accounts for ~20–30% of NSCLCs (3). Compared with lung adenocarcinoma (ADC), there are no effective therapeutic strategies for SCC, which is associated with fewer known somatic mutations vulnerable to targeted therapy (e.g., EGFR mutations, ALK rearrangement) (4, 5). Furthermore, SCCs are characterized as highly aggressive tumors, and patients with SCC frequently show resistance to conventional chemotherapy, which is associated with their poor prognosis (6). Therefore, it is urgent to explore a novel and effective therapeutic strategy for lung SCC patients.

Compared with lung ADCs, lung SCCs have higher levels of glucose metabolism to meet the anabolic and catabolic needs required to sustain rapid tumor growth (7). Hexokinases (HKs) catalyze the rate-limit and first committed step in glucose metabolism by phosphorylating glucose (8, 9). There are five main hexokinases in mammalian cells, including HK1–4 and the recently identified hexokinase domain-containing protein 1 (HKDC1) (10–12). Particularly, HK1 and HK2 preferentially use ATP derived from the mitochondria to phosphorylate glucose, thereby coupling oxidative phosphorylation(OXPHO) with glycolysis (13). HK3 has the highest affinity for glucose but is also inhibited by glucose at physiological levels, and its precise role in glucose metabolism in cancer cells is not clear. By contrast, HK4 has low affinity for glucose and is not inhibited by G6P (10, 11, 13). However, unlike HK1, HK2 shows high expression to enhance glucose flux into various metabolic pathways in most cancer cells (14, 15). However, whether HK2 could be used as a novel and potential therapeutic target for lung SCC is still a mystery.

AMP-activated protein kinase (AMPK) is a sensor of cellular energy and nutrient status, which can inhibit its downstream PI3K–AKT–mTOR complex 1 (mTORC1) signaling pathway. PI3K–AKT–mTORC1 signaling has an evolutionarily conserved function in metabolism and is also frequently activated in cancer cells (16). Importantly, the PI3K-AKT-mTOR signaling axis is a druggable pathway as evidenced by multiple early-phase clinical trials in tumor therapy (7). Lung SCC cells have the ability to bypass mTOR inhibition and the suppression of glycolysis by upregulating glutamine metabolism as a compensatory mechanism to deal with energy stress (7). Besides, OXPHO was elevated as a consequence of glycolytic inhibition in hepatocellular carcinoma, which maintained the TCA cycle and energy for tumor cell growth (17). This suggests that multi-pathway inhibition combination therapy is required to develop an effective treatment for cancer. Metformin, a first-line drug for treating type 2 diabetes, showed a potential for tumor treatment owing to its glucose-lowering effect (18) and functioned as a specific inhibitor of the mitochondrial respiratory-chain complex 1. In addition, metformin could reduce the generation of reactive oxygen species at the complex 1 and prevent mitochondrial-mediated apoptosis, suggesting its potential protective effect against oxidative stress-induced cell death (19).

In this study, we found that HK2 is highly expressed in lung SCC, but not in lung ADC, and is required for tumor growth. Moreover, screening of public databases from The Cancer Genome Atlas (TCGA) and Gene Expression Omnibus (GEO) led us to further focus on the AMPK-mTORC1 pathway in lung SCC patients with high HK2 expression. In addition, silencing HK2 induced oxidative phosphorylation as a compensatory mechanism. Therefore, we examined the cancer cell-killing effect of the combination of HK2 ablation and metformin *in vitro* and *in vivo*. The combination of HK2 depletion and metformin treatment could significantly induce lung SCC apoptosis, providing a scientific foundation for further exploration of a new therapeutic strategy to improve the outcome and quality of life of patients with lung SCC.

## Materials and Methods

### Cell Lines and Cell Culture

The H226 cells were cultured in 1640 medium supplemented with 10% bovine pituitary extract. The H520 cells, A549 cells and PC9 cells were cultured in DMEM medium supplemented with 10% bovine pituitary extract. NSCLC cell lines were obtained from the American Type Culture Collection (ATCC) and were tested and authenticated by DNA typing at Shanghai Jiao Tong University Analysis Core. These cells were all cultured at 37°C in a humidified incubator in an atmosphere of 95% air and 5% CO_2_.

### Western Blotting

All samples were lysed in RIPA buffer and separated by SDS-PAGE and transferred to NC membranes (GE Healthcare Life science, Lot. G9597136). The 5% (w/v) no-fat dry milk in TBST was used to block non-specific binding sites. The blocked membranes were incubated with the specific primary antibodies diluted in 5%BSA (Sigma-Aldrich) with 0.05% sodium azide for overnight on shacking bed at 4°C. HRP-conjugated secondary antibody was incubated at room temperature for 1 h. Finally, the membranes were visualized by exposure with Immobilon Western Reagents (Millipore, Lot.15015B4).

### Soft Agarose

Add higher concentration agarose (1:3, final concentration:0.7%) to 24-well (0.5 ml per well), then put H226 si-NC cells and H226 si-HK2 cells (500 cells in 50 μl medium) on the agarose. On the top, add lower concentration agarose (1:6, final concentration: 0.35%) with matrigel (1:30) on the cells. In metformin treating group, add 5 mM metformin in the top mixture. The plate was incubated in 37°C/5% CO_2_ incubator, which was taken a picture and statistics the colony numbers after 10 days.

### 2-Dimensional Colony Formation Assay

H226-siNC/HK2 cells and H520-siNC/HK2 cells were feed in 6 cm dish (400 cells). Change the medium every 3 days. After 2 weeks, the cell was stained with 1% Methylrosanilnium Chloride Solution for 15 min.

### Transfection and Stably Transfected Cells

H226 cells and H520 cells were transfected with siRNA-NC(AGGTAGCTGCCTAGAGCAG) and siRNA-HK2 1#-3# (1#:AAGCTACAAATCAAAGACA, 2#:CTCTTTAACCTCATCTACA, 3#:GCACTGATGTTGTTACTGA)through Lipo 2000 (plasmid: Lipo2000, 1:1) transfection reagents, which replaced medium after 4–6 h. Then, these cells were cultured for 72 h and lysis the protein for detecting the efficiency of silence.

### Tumorigenicity in Nude Mice

Eight-week-old nude mice were injected with H226-si-NC/HK2 (5 × 10^6^ cells) combined with Matrigel (*n* = 6 per group). One week after injection, the mice were gavage with metformin (250 mg/kg), they were treated daily for 3 weeks. Two weeks later, the volume of tumors were measured and further analyzed. All mice were maintained according to a protocol approved by Shanghai Jiao Tong University, School of Medicine Animal Care and Use Committee [experimental animal use permission No: SYXK (Shanghai) 2008-0050] in the specific pathogen-free animal facility in the university.

### Immunohistochemistry (IHC)

A tissue microarray composed of ADC and SCC tissues were stained to identify HK2 proteins. The IHC protocol and score method were performed as previously described (20). All antibodies were diluted for use according to manufacturers' instructions.

### Reagents and Antibodies

#### Reagents

Lonidamine (S2610) was from Selleck.cn. BSA (V900933) and Dulbecco′s Modified Eagle′s Medium-high glucose (D0422-100ML) were from Sigma-Aldrich. Matrigel Matrix (354262) was from Corning. Annexin V-APC/PI Apoptosis Detection Kit (KGA1030-50) was from KeyGEN BioTECH. Glucose Colorimetric Assay Kit (K686-100) and Lactate Colorimetric Assay Kit (K627-100) were from BioVision. Mito Stress Test Kit (103015-100) was from Agilent.

#### Antibodies

Anti-phospho-AMPK (Thr172) antibody (#2535S), Anti-AMPKα Antibody (#2532), anti-p70-S6K (9202S), anti-phospo-p70-S6K (Thr389) (9234S), anti-Hexokinase 2 (2867S), anti-phospho-4EBP1 (Thr70) (13396) and anti-4EBP1 (9644s) were from Cell Signaling Technology. Anti β-actin-HRP (PM053-7) from MBL. Cleaved PARP antibody (#5625) was from Cell Signaling Technology. The antibodies were diluted according to manufacturers' instructions.

### Apoptosis Assay by FACS

The Annexin V-APC/PI Apoptosis Detection Kit was used to analyze the apoptosis in H226 cells. H226 siNC and H226 siHK2 cells (4 × 10^5^ cells/mL) were suspended in 200 μl PBS, then adding 5 μl annexin V and 5 μl PI reagents into cell suspension. Aliquots were incubated for 15 min at room temperature protected from light. The gates were established using the negative controls for compensation. Finally, the data was analyzed in the FlowJo 7.6.1 software.

### Seahorse XF24 Respirometry

The seahorse assay was measured as previously described (21). The Oxygen Consumption Rate (OCR) and Extracellular Acidification Rate (ECAR) were measured using a Mito Stress Test Kit from Agilent and XF24 Extracellular Flux Analyzer (Seahorse Bioscience) according to the manufacturer's protocol. In brief, 150,000 cells were plated in 100 μL of their standard growing media and cultured overnight. The day of the measurement, cells were washed with XF media (1% FBS) and incubated in a CO_2_-free incubator at 37°C for 2 h to establish equilibration prior to loading. Basal conditions consisted of XF media in 1% FBS before the addition of 10 mM glucose. ECAR and OCR measurements were taken before and after addition of glucose (10 mM), oligomycin (1 μM), Rotenone/Myxothiozol (0.5 μM each), FCCP (1 μM) and Monensin (20 μM) and used to calculate ATP production, bioenergetic capacity and supply flexibility index as previously described (22).

### Glucose Uptake and Lactate Production

The level of glucose and lactate in the H226 cells were detected using the Glucose Colorimetric Assay Kit and the Lactate Colorimetric Assay Kit, and performed according to the methods provided in the kit.

### Bioinformatics Analysis

We gain the data from TCGA, GEO and TCPA database. Firstly, we download the raw data that we interesting from these websites. Then we selected the expression data of HK2 and survival information. Using the GeneSpring software from Agilent, we analysis the different genes and signaling pathways in a certain rules (fold change > 2, *p* < 0.05), for example, HK2-high expression verse HK2-low expression (cut-off value: average value). Finally, we put these selected data including up-regulated genes and down-regulated genes to DAVID website for KEGG pathway analysis.

### Statistical Analyses

**Figure 1:**
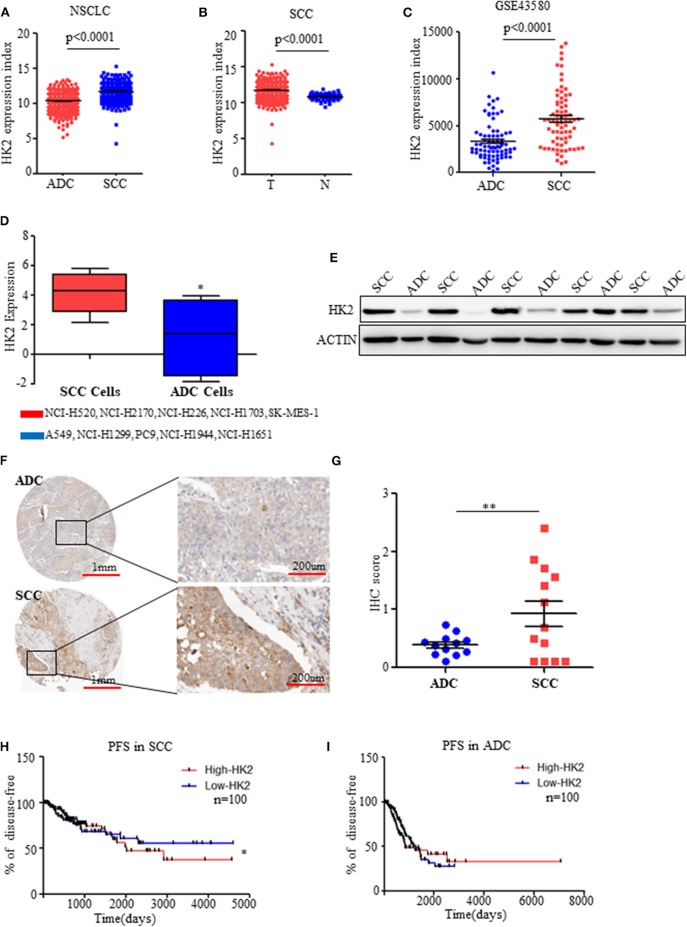
HK2 is highly expressed in SCC compared to ADC. **(A)** HK2 expression index in NSCLC samples from TCGA database. **(B)** HK2 expression index in SCC tumor samples compared with that in normal tissues from TCGA database. **(C)** HK2 expression index in NSCLC from the GSE43580 cohort. **(D)** HK2 expression in SCC cell lines (NCI-H520, NCI-H2170, NCI-H226, NCI-H1703, SK-MES-1) and ADC cell lines (A549, NCI-H1299, PC9, NCI-H1944, NCI-H1651). **(E)** Western blot showing the expression of HK2 in lung SCC (*n* = 6) and ADC (*n* = 6) samples. **(F)** Representative image of immunohistochemical (IHC) staining for HK2 expression in SCC (*n* = 12) and ADC (*n* = 11) patients. **(G)** IHC scores to quantify the expression of HK2 in ADC and SCC samples. **(H)** Percentage of PFS in SCC patients according to HK2 expression (High-HK2 *n* = 100, Low-HK2 *n* = 100) in TCGA database. **(I)** Percentage of PFS in ADC patients according to HK2 expression (High-HK2 *n* = 100, Low-HK2 *n* = 100) in TCGA database. *p < 0.05, **p < 0.01.

Data were analyzed using the software SPSS Statistics (IBM, Version 19). Data were presented as the mean ± standard deviation. The differences of results were compared using two-tailed nonparametric Mann-Whitney test, confidence Interval 95% [the expression of HK2 in ADC and SCC (Figures 1A–C)]. The differences of results were compared using two-tailed unpaired *t*-test, Confidence Intervals 95% (the expression of HK2 in cell lines, the IHC score, the colony number difference). The statistical test for the Kaplan-Meier survival curve data was using Log-rank (Mantel-Cox) test analysis. *p*-value < 0.05 was considered statistically significant.

## Results

### HK2 Is Highly Expressed in SCC Compared to ADC

Using data on NSCLC patients from the TCGA database (23, 24), HK2 expression was significantly higher in SCCs (*n* = 502) than in ADCs (*n* = 515) (Figure 1A). Interestingly, the expression of HK2 was only high in SCC tumors (*n* = 502) compared with that in adjacent normal tissues (*n* = 51) (Figure 1B). There was no such difference in total NSCLC patients (tumor *n* = 1,017, normal *n* = 110), and normal tissues (*n* = 59) showed higher HK2 expression than tumor tissues (*n* = 515) from ADC patients (Supplementary Figures 1A,B). Similarly, HK2 showed increased expression in the SCC (*n* = 73) cohort compared to that in the ADC (*n* = 77) cohort in the GEO database GSE43580 (25) (Figure 1C).

Consistent with these results from patient samples, information on various SCC and ADC cell lines from the TCPA website (http://tcpaportal.org/) demonstrated that the expression level of HK2 was increased in SCC cells(NCI-H520, NCI-H2170, NCI-H226, NCI-H1703, SK-MES-1) compared with that in ADC cells(A549, NCI-H1299, PC9, NCI-H1944, NCI-H1651) (Figure 1D). Western blot analysis of tumor samples collected from patients with NSCLC further showed significantly increased protein expression levels of HK2 in SCC patients compared with levels in ADC patients (Figure 1E), which was confirmed by staining data of the expression of HK2 from the THPA database (www.proteinatlas.org/) (Figures 1F,G).

Patients in the TCGA database were divided into two groups: high and low HK2 expression groups, and survival analysis was performed. The results indicated that patients with high HK2 expression (*n* = 100) had worse Progression Free Survival (RFS) than those with low HK2 expression (*n* = 100) (Figure 1H). However, this effect was only observed in SCC patients, but not in ADC patients or total NSCLC patients, for both PFS and overall survival (Figure 1I and Supplementary Figures 1C–F). Overall, these results suggested that HK2 expression is specifically elevated in SCC.

### HK2 Contributes to Lung Squamous Tumor Cell Growth

**Figure 2:**
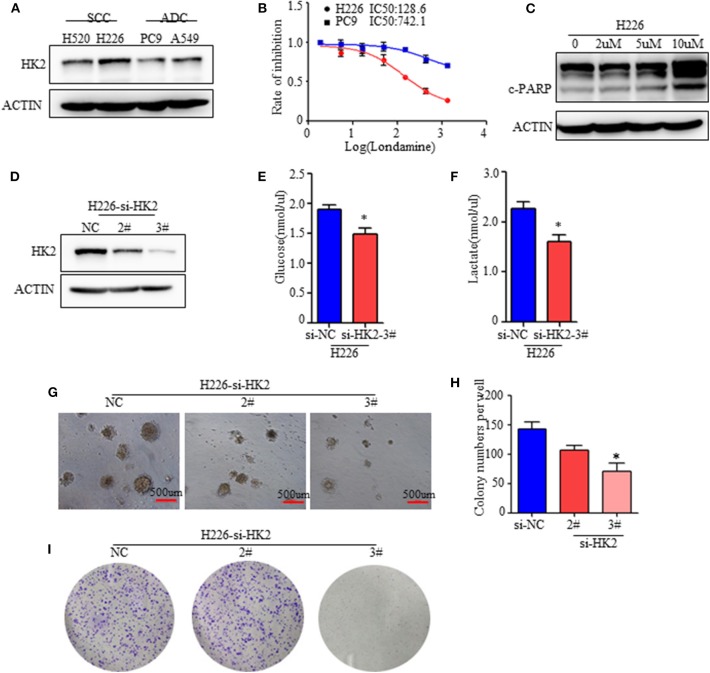
HK2 contributes to lung squamous tumor cell growth. **(A)** Western blot showing the expression of HK2 in SCC and ADC cell lines. **(B)** Rate of inhibition in H226 and PC9 cells with or without lonidamine treatment. **(C)** Expression of cleaved-PARP in H226 cells treated with different doses of lonidamine. **(D)** Western blot showing the efficiency of silencing HK2 in the H226 cell line. **(E,F)** Glucose uptake and lactate production in H226 si-NC- and si-HK2-transfected cells using the Glucose and Lactate Colorimetric Assay Kit. **(G)** Colony formation assay showing anchorage-independent growth in H226 cells. **(H)** Colony formation numbers in H226-si-NC- and H226-si-HK2-transfected cells. **(I)** 2-dimensional colony formation assay of H226-siNC and H226-siHK2 cells. *p < 0.05.

To clarify the effect of HK2 on SCC, we conducted *in vitro* experiments with two lung ADC cell lines (A549 and PC9) and two lung SCC cell lines (H520 and H226) reported to have clear differences in HK2 expression (Figure 1D). We confirmed the increased expression of HK2 in the SCC cell lines (Figure 2A). Moreover, H226 and H520 cells showed greater sensitivity to treatment with lonidamine, a small-molecule hexokinase inhibitor, in terms of inhibition of cell proliferation, than did PC9 and A549 cells (Figure 2B and Supplementary Figure 2A). Further analysis showed that this inhibition was accompanied by increased expression of the apoptosis marker, cleaved PARP, indicating that the mechanism of HK2 inhibition by lonidamine involves the induction of tumor cell apoptosis (Figure 2C and Supplementary Figure 2B).

To further determine the specific function of HK2 in lung tumors, especially in SCC, we silenced *HK2* gene expression via transfection of H226 cells with small interfering RNA (siRNA) targeting *HK2*. Western blotting confirmed the efficiency of HK2 silencing after transfection (Figure 2D). HK2-silenced H226 cells took up less glucose and produced less lactate than control si-NC-transfected cells (Figures 2E,F). In addition, HK2silencingsignificantly reduced anchorage independent growth in H226 cells (Figures 2G,H). In addition, the plate colony formation assay showed decreased cell proliferation in H226-siHK2 cell lines, especially in H226-siHK2-3# (Figure 2I). Similar results were obtained for H520, another SCC cell line (Supplementary Figure 2C). Taken together, these findings demonstrated that HK2 is required for tumor glycolysis and lung squamous cell tumorigenesis.

### Inhibiting HK2 Induces Activation of the AMPK Signaling Pathway

**Figure 3:**
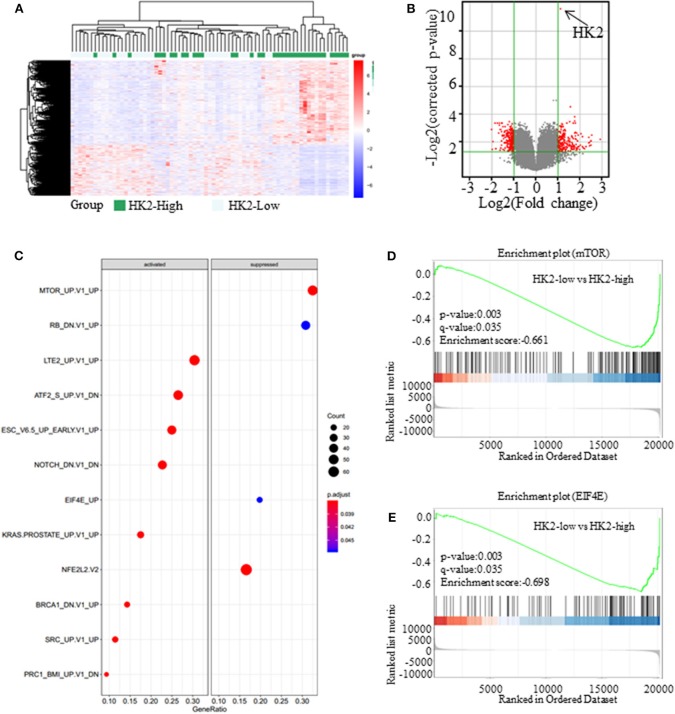
Inhibiting HK2 induces activation of the AMPK signaling pathway. **(A)** The expression of different genes in High-HK2 and Low-HK2 samples from the GSE43580 cohort. **(B)** Volcano map showing the differentially expressed genes (fold change > 2, *p* < 0.05, red dots are up-regulated genes and down-regulated genes). **(C)** GSEA shows the differential signaling pathways enriched from differential genes. **(D)** Running enrichment score of mTOR in GSEA. **(E)** Running enrichment score of mTOR in GSEA.

Besides its role in glucose anabolism, we sought to determine the effect of HK2 on tumor proliferation and the underlying mechanism. For this purpose, SCC patients (*n* = 73) were selected from the GSE43580 cohort and divided into two groups according to the high or low expression of HK2. Differential gene expression in HK2-High and HK2-Low patients is shown in Figure 3A (Supplementary Data 1). Bioinformatics analysis with GeneSpring GX software was used to determine the influence of HK2 expression on the general gene expression profile in these groups (Supplementary Figure 3A). Principal components analysis was then used to reduce the dimensionality of the data and transform multiple indicators of the differentially expressed genes into three comprehensive indicators for more targeted analysis (Supplementary Figure 3B). We ultimately identified 228 up-regulated genes and 224 down-regulated genes (fold change > 2 and *p* < 0.05) in the HK2 high-expression group compared to those in the HK2 low-expression group (Figure 3B). Through the GSEA (Gene Set Enrichment Analysis), we focused on the down-regulated pathways, mTOR and EIF4E, in HK2-Low patients (Figures 3C–E). Besides, these differentially expressed genes were subjected to Kyoto Encyclopedia of Genes and Genomes pathway analysis for functional annotation using DAVID tools (https://david.ncifcrf.gov/), which revealed an important role of AMPK-related pathways, including significant downregulation of the cGMP-PKG, RAS, and PI3K-AKT signaling pathways in the HK2 high expression group (Supplementary Figures 3C,D). Therefore, we hypothesized that inhibiting HK2 induces activation of the AMPK signaling pathway and inhibition of the mTOR pathway, which could be related to the increased energy stress in lung squamous tumors.

### Inhibiting HK2 Activates AMPK and Subsequently Inhibits mTORC1 Activity

**Figure 4:**
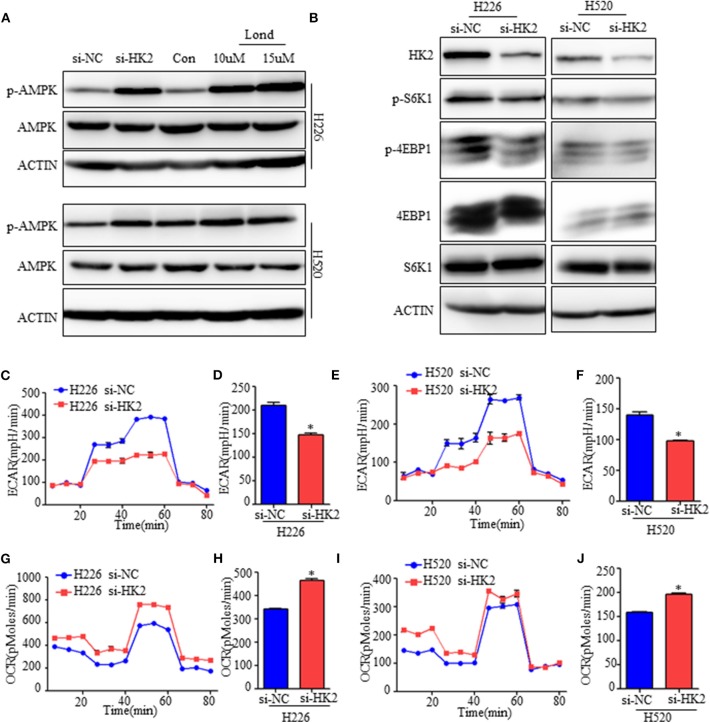
Inhibiting HK2 activates AMPK and subsequently inhibits mTORC1 activity. **(A)** Western blot showing the expression of phospho-AMPK in H226 si-HK2- and H520 si-HK2-transfected cells compared with that in siNC-transfected cells or cells treated with different doses of lonidamine. **(B)** Immunoblot showing the expression of phosphor-S6K1 and phosphor-4EBP1 in H226 siHK2- and H520 si-HK2-transfected cells compared with that in H226 siNC- and H520 siNC-transfected cells, respectively. **(C,D)** Seahorse metabolic analysis of the extracellular acidification rate (ECAR) in H226 si-NC- and siHK226-transfected cells. **(E,F)** Seahorse metabolic analysis of ECAR in H520 si-NC- and siHK520-transfected cells. **(G,H)** Seahorse metabolic analysis of oxygen consumption rates (OCRs) in H226 si-NC- and siHK226-transfected cells. **(I,J)** Seahorse metabolic analysis of OCRsin H520 si-NC- and siHK520-transfected cells. *p < 0.05.

Tumor cells under energy stress will activate the AMPK pathway as a compensatory response. HK2 depletion or inhibition have been shown to decrease aerobic glycolysis of SCC, so we detected the expression of the phosphorylation level of AMPK (Thr172), the activate form of AMPK pathway. As shown, the AMPK pathway was significantly activated neither HK2 silencing nor its inhibition with lonidamine (Figure 4A). Since energy stress-induced activation of AMPK is also known to inhibit the activation of mTORC1, we next examined the effect of HK2 silencing on mTORC1 activity. Western blotting results suggested that HK2 depletion significantly decreased the activity of the mTORC1 pathway as indicated by the level of S6K1 (Thr389) and 4EBP1 (Thr70) phosphorylation (Figure 4B).

To further understand the mechanism, we examined the metabolic consequences of HK2 deficiency using the Seahorse metabolic analyzer. The extracellular acidification rate (ECAR) was significantly reduced in both H226-siHK2- and H520-siHK2-transfected cells compared with that in control cells, indicating suppression of glycolytic function (Figures 4C–F). We hypothesized that the energy production derived from glycolysis decreased in HK2-silenced cells, resulting in increased basal respiration and mitochondrial efficiency as a compensatory adaptive response of the cells to balance energy requirements. Indeed, analysis of the respiration rate following HK2 silencing showed increased oxygen consumption rates (OCRs) and respiration in H226-siHK2-transfected cells compared with those in control cells (Figures 4G,H), and the same tendency was found for H520-siHK2-transfected cells (Figures 4I, J). Taken together, these data indicate that energy stress in HK2-silenced lung squamous cells activates AMPK to subsequently inhibit mTORC1 activity.

### Effect of HK2silencing and Metformin Treatment on mTORC1 Activity

**Figure 5:**
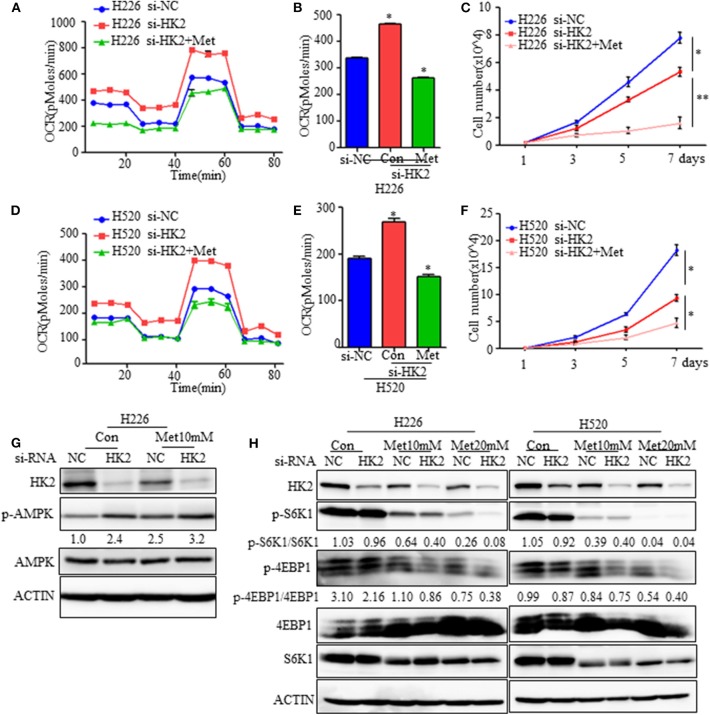
Effect of HK2silencing and metformin treatment on mTORC1 activity. **(A,B)** Seahorse metabolic analysis of oxygen consumption rates (OCRs) in H226 si-NC- and siHK226-transfected cells with or without metformin treatment (10 mM). **(C)** Cell number representing the proliferation of H226 siNC- and siHK2-transfected cells with or without metformin treatment (10 mM). **(D,E)** Seahorse metabolic analysis of oxygen consumption rates (OCRs) in H520 si-NC- and siHK2-transfected cells with or without metformin treatment (10 mM). **(F)** Cell number representing the proliferation of H520 siNC- and siHK2-transfected cells with or without metformin treatment (10 mM). **(G)** Immunoblot showing the expression of phospho-AMPK. **(H)** Immunoblot showing the expression of phosphor-S6K1 and phosphor-4EBP1 in H226/H520 siNC- and siHK2-transfected cells with or without treatment with different doses of metformin. *p < 0.05, **p < 0.01.

Although HK2 contributes to lung squamous tumor proliferation *in vitro*, information on its effects on tumorigenesis is limited. Since silencing HK2 reduced the rate of glycolysis and up-regulated oxidative phosphorylation as a compensatory mechanism, we hypothesized that targeting both metabolic pathways may be a potentially effective therapeutic strategy for SCC. To test this idea, we employed metformin, a first-line drug for type 2 diabetes and a complex I inhibitor (26, 27), to decrease the raised oxidative respiration in H226-siHK2-transfected cells. As shown in Figures 5A,B, the increase in oxygen consumption was diminished after treatment with metformin in conjunction with HK2 silencing. The same experimental results were obtained for H520 cells (Figures 5D,E). We also detected the effect of the combination of metformin and HK2 silencing on tumor cell proliferation. Consistently, the proliferation of H226-siHK2-transfected cells and H520-siHK2-transfected cells treated with metformin was significantly inhibited compared with that of H226-siNC-transfected cells and H520-siNC-transfected cells, respectively (Figures 5C–F).

Since metformin treatment of HK2-silenced cells leads to greater energy stress, we further examined the effects of combined HK2 depletion and metformin treatment on AMPK and mTORC1 activities. As expected, the AMPK pathway was activated either HK2 silencing or metformin treatment alone, and the activation becomes more pronounced in the combined application of HK2 silencing and metformin treatment (Figure 5G). However, mTORC1 activity, as measured by phosphorylated S6K (Thr389) and 4EBP1 (Thr70) levels, was more strongly diminished in H226-siHK2-transfected cells treated with metformin, and this inhibition effect was dose-dependent (Figure 5H). Similar results were obtained for H520 cells (Figure 5H). These findings suggest that metformin treatment could offset the increased compensatory effect in oxidative respiration in HK2-silenced tumors.

### Effect of HK2 Knockdown and Metformin on Cell Death and Tumor Growth

**Figure 6:**
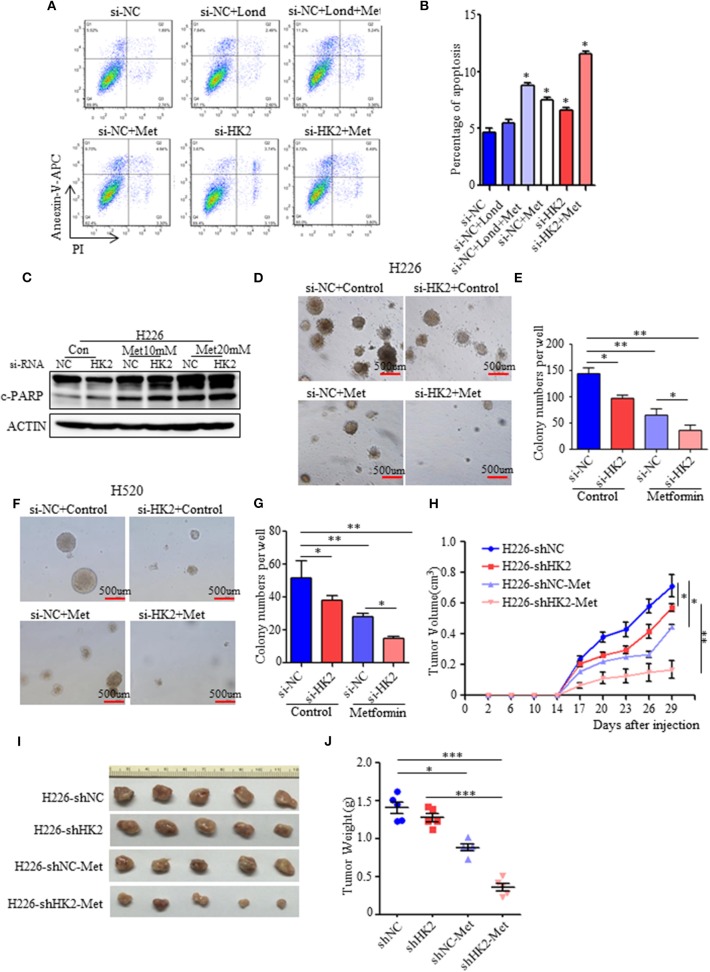
Effect of HK2 knockdown and metformin on cell death and tumor growth. **(A,B)** The effect of HK2silencing or treatment with lonidamine in combination with metformin (10 mM) on death of H226 cells. **(C)** Immunoblot showing the expression of cleaved PARP in H226 siNC- and H226 siHK2-transfected cells with or without treatment of different doses of metformin. **(D)** Colony formation assay showing the anchorage-independent growth in H226 si-NC-transfected cells compared with that in H226 si-HK2-transfected cells with or without metformin treatment. **(E)** The colony formation numbers in H226 si-NC- and H226 si-HK2-transfected cells with or without metformin treatment. **(F)** Colony formation assay showing the anchorage-independent growth in H520 si-NC-transfected cells compared with that in H520 si-HK2-transfected cells with or without metformin treatment. **(G)** The colony formation numbers in H520 si-NC- and H520 si-HK2-transfected cells with or without metformin treatment. **(H)** Tumor volumes were assessed in A226-siNC/siHK2-3# cells with or without treatment of metformin. Nude mice were injected with cells (5 × 10^6^ cells, *n* = 5), cells were mixed in Matrigel, and tumor volumes of the different treatment groups were measured as indicated. **(I)** Tumor pictures are shown for A226-siNS/siHK2-3# cells with or without treatment of metformin. **(J)** Tumor weight was assessed in A226-siNC/siHK2-3# cells with or without treatment of metformin. *p < 0.05, **p < 0.01, ***p < 0.001.

Although basal respiration and mitochondrial efficiency increased as a compensatory adaptation to energy stress resulting from HK2 depletion, this was accompanied by partial cell death and apoptosis due to the decrease in glycolysis. Since metformin treatment could diminish the enhanced oxygen consumption, we hypothesized that HK2 silencing combined with metformin could further enhance the rate of tumor cell apoptosis. Indeed, flow cytometry showed that the rate of tumor cell apoptosis did increase with either HK2 silencing or metformin treatment alone, or in combination, and increased more by treatment of both lonidamine and metformin (Figures 6A,B). Similar results were obtained for H520 cells (Supplementary Figure 4). We also evaluated apoptosis induction by detecting the expression of the apoptosis marker, cleaved PARP, using western blot analysis. As shown in Figure 6C, the expression level of cleaved PARP increased under HK2 depletion alone or under the combination of HK2 depletion and metformin treatment. However, a soft agarose assay showed the synergistic effects of metformin and HK2 silencing on anchorage-independent growth of tumor cells. HK2 depletion or metformin alone also significantly inhibited colony formation; importantly, the combination of metformin and HK2 depletion decreased tumor growth much more substantially than either treatment alone (Figures 6D–G). Furthermore, we examined the tumorigenicity (*n* = 6 per group) of these cells *in vivo* and found that the tumorigenicity of H226-siHK2 and H226-siNC-Met was significantly decreased compared with that of control H226-siNS cells. Interestingly, when H226-siHK2 cells were treated with metformin alone, the tumor volume and weight were significantly lower than those in the other groups (Figures 6H–J). Taken together, the combination of HK2 depletion and metformin treatment could lead to more tumor cell apoptosis and reduce tumor growth.

## Discussion

**Figure 7:**
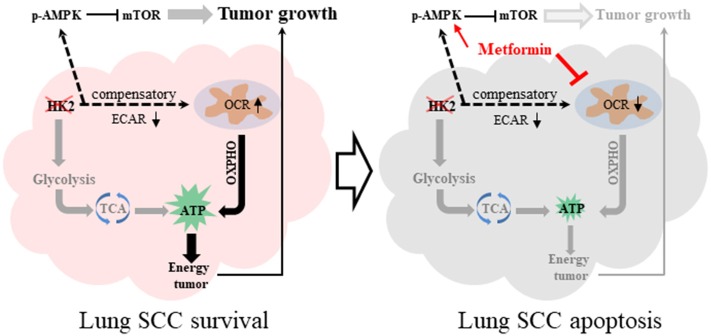
Graphical summary. HK2 depletion leads to oxidative phosphorylation compensatory increase. The combination therapy of HK2 depletion and metformin treatment leads to more lung SCC apoptosis.

This study demonstrates that HK2, a key enzyme of glycolysis, is highly expressed in lung SCC, but not in ADC, and is critical for cell proliferation. Further investigation demonstrated that depletion of HK2 activated AMPK to subsequently inhibit mTORC1 activity, which plays a sensor role in cancer metabolism. Finally, we showed that the combination of HK2 ablation and metformin treatment, which decreased the compensatory up-regulated oxygen respiration under HK2 depletion, could enhance lung SCC cell death and inhibit tumor cell growth (Figure 7). Given the lack of treatment options for patients with lung SCC, these data could offer a new direction for targeted therapeutic strategies.

Most cancer cells, especially lung SCC cells, reprogram cellular glucose metabolism to fulfill their high anabolic demands. In contrast to ADCs, there is a lack of known targetable driver mutations in SCCs, despite the high mutational burden and diverse mutational landscape (28, 29). Thus, as a starting point to develop an effective targeted therapy for SCC, we focused on the glycolytic tumor characteristics to identify a mechanism for devising a strategy of jointly inhibiting tumor metabolic pathways for treating cancer. Here, we used lonidamine to inhibit the enzyme activity of HK2 in SCC. In normal and tumor cells, lonidamine reduces oxygen consumption, while it increases aerobic glycolysis in normal cells and inhibits aerobic glycolysis in tumor cells (30). HK2 was the key enzyme in SCC aerobic glycolysis, so the lonidamine could also reduce the enzyme activity of hexokinase in SCC. Moreover, tumor cells show a high degree of plasticity, and thus it will be difficult to suppress tumor growth by inhibiting a single pathway. Metabolic profiling of lung SCC tissues using positron emission tomography and liquid chromatography-mass spectrometry identified a dual reliance on both glutamine and glucose metabolism. Importantly, glutamine metabolism was shown to be altered in an adaptive manner in lung SCC following chronic mTOR inhibition, which could explain the decrease in glycolysis observed in patients (7, 31, 32). Further investigation identified the GSKα/β pathway as a central regulator of this adaptive glutamine metabolism that enables lung SCC tumors to escape the effects of mTOR inhibition by upregulating glutaminase expression (7). Thus, the present work further reflects the plasticity of SCC tumors; that is, when blocking a classic metabolic pathway, another metabolic pathway is activated as a compensatory mechanism to maintain the growth of the tumor cells. In this study, oxidative phosphorylation could be inhibited by metformin in lung SCC, providing an effective therapeutic strategy. Another recent study demonstrated that HK2 deficiency markedly increased the susceptibility of hepatocellular carcinoma cells to the FDA-approved drug, sorafenib, and also enhanced the inhibitory effects of sorafenib on tumor growth *in vivo* (17). These findings offer a new perspective for tumor treatment. Blocking tumor-related signaling pathways will have indirect or direct effects on the metabolism of tumors, which may be a powerful link for suppressing tumor growth.

The success of cancer therapeutics in clinical practice relies on gaining a comprehensive understanding of the underlying mechanism of action. Deregulated tumor cell metabolism is recognized as one of the hallmarks of cancer. This not only reflects the complicated metabolic environment in cancer cells (33) but also provides a good opportunity for treatment, with more options as targets. As a proof-of-concept, pan-cancer metabolism analysis-based prediction of metabolic gene expression signatures can help to select a tumor that is sensitized to a metabolic inhibitor using large-scale cell line profiling and robust bioinformatics methods (34). Our study provides a framework for understanding the basis of HK2 dependency in lung SCC tumors and further suggests a novel therapeutic rationale for combing HK2 depletion with metformin treatment to suppress oxygen respiration to place cancer cells in a greater state of energy stress to suppress growth.

## Data Availability Statement

All datasets generated for this study are included in the article/Supplementary Material.

## Ethics Statement

The animal study was reviewed andapproved by Shanghai Jiao Tong University, School of Medicine Animal Care and Use Committee [experimental animal use permission No: SYXK (Shanghai) 2008-0050] in the specific pathogen-free animal facility in the university.

## Author Contributions

WG, YK, JW, DW, QW, and WW contributed to the experiment design, manuscript draft, and data analysis. AZ, HS, YL, DX, TW, BJ, KL, and JL performed the experiments. MH performed the bioinformatics analysis.

### Conflict of Interest

The authors declare that the research was conducted in the absence of any commercial or financial relationships that could be construed as a potential conflict of interest.

## Supplementary Material

Supplementary Figure S1 HK2 is high expression in SCC compared to ADC.

Supplementary Figure S2 HK2 is required for lung squamous tumorigenesis.

Supplementary Figure S3 Inhibiting HK2 induces the activation of AMPK signaling pathway.

Supplementary Figure S4 Effect of HK2 knockdown and metformin on cell death and tumor growth.

Supplementary Data 1 Different Gene (fold>2 *p*-value<0.05).
