# Supplementary figures and images for: Hexokinase 2 Depletion Confers Sensitization to Metformin and Inhibits Glycolysis in Lung Squamous Cell Carcinoma
Source: Front Oncol. 2020 Jan 31;10:52. doi: 10.3389/fonc.2020.00052 (PMC7005048; doi:10.3389/fonc.2020.00052)

Supplementary Figure 1

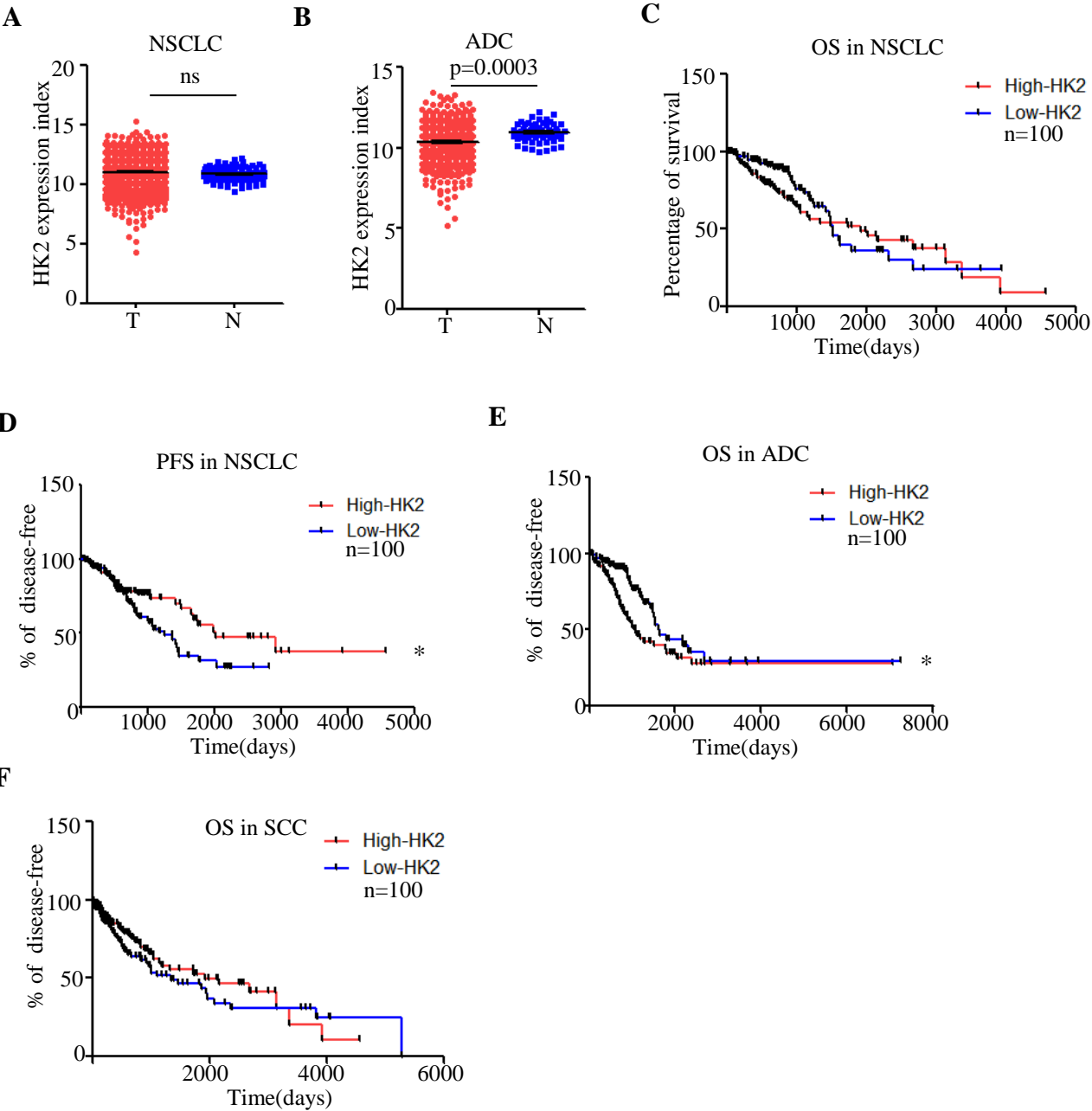

Supplementary Figure 2

A

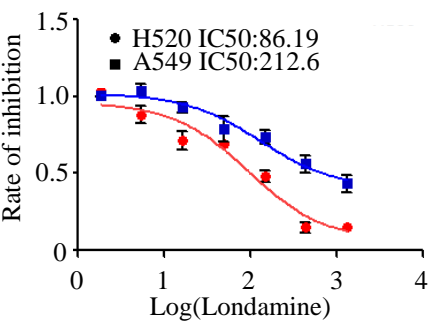

B

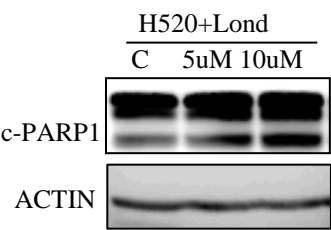

C

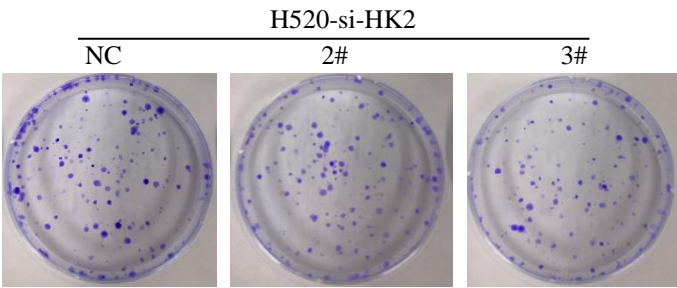

Supplementary Figure 3

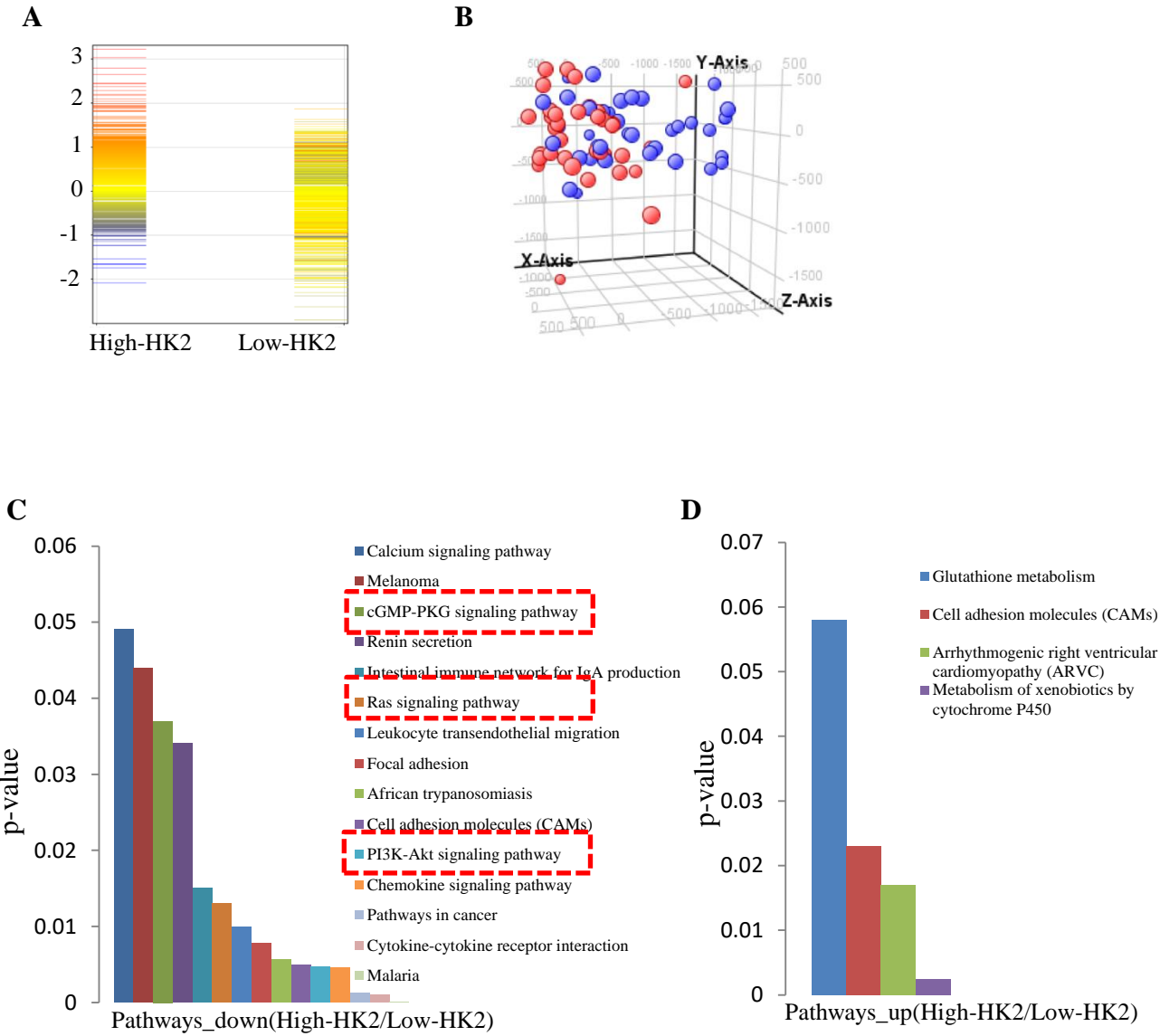

Supplementary Figure 4

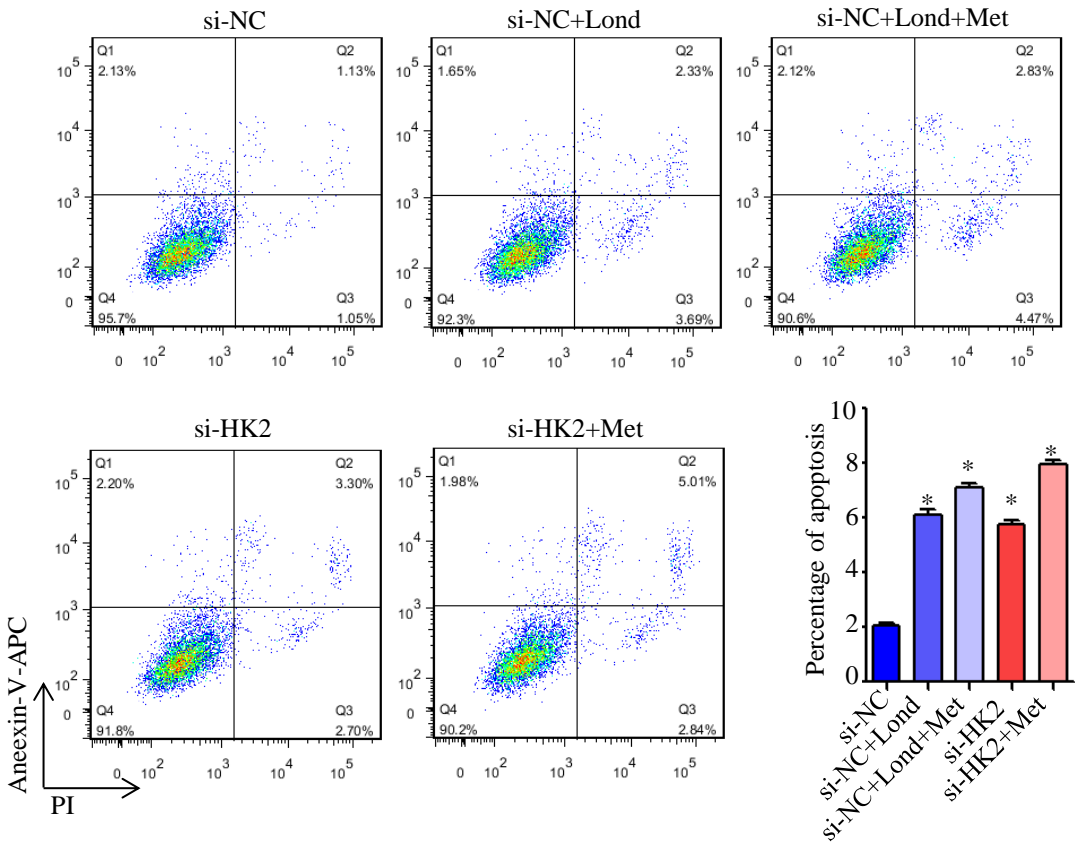

Supplement: Supplementary Figure S1 — HK2 is high expression in SCC compared to ADC. [file Data_Sheet_1.PDF]
